# Preliminary investigation on the economic cost of mitochondrial disease in Chinese children

**DOI:** 10.1186/s13023-025-03708-1

**Published:** 2025-04-10

**Authors:** Chaolong Xu, Dan Zhao, Xin Duan, Zhimei Liu, Tongyue Li, Yunxi Zhang, Zixuan Zhang, Tianyu Song, Ying Zou, Huafang Jiang, Fang Fang

**Affiliations:** 1 Department of Neurology, Beijing Children’s Hospital, Capital Medical University, National Center for Children’s Health, Beijing, China; 2 Department of Pediatrics, WeiFang Maternal and Child Health Hospital, Weifang, China

**Keywords:** China, Children, Economic costs, Mitochondrial disease

## Abstract

**Background:**

The prevalence of mitochondrial diseases is increasing, leading to a significant economic burden on families and society. However, nationwide cost data on their effects on China’s economy remain limited. This study aimed to investigate the economic cost of mitochondrial diseases in Chinese children, analyse the relevant influencing factors, and provide a foundation for strategies to reduce the healthcare burden.

**Methods:**

In this single-centre, cross-sectional study, an online questionnaire was randomly administered to paediatric patients diagnosed with mitochondrial diseases between January 2012 and January 2022. The questionnaire included questions regarding demographic data, clinical information, and expenditure-related costs. Multivariate analysis of economic cost was performed using a generalised linear gamma conjugate model (A1).

**Results:**

The responses to 102 questionnaires were analysed. The median direct economic cost incurred for the diagnosis of mitochondrial disease was $8,520.19, with direct medical and non-medical costs of $6,769.06 and $2,092.98, respectively, and an indirect cost of $3,162.93. Healthcare insurance covers 27.29% of direct medical expenses. Multivariate analysis showed that the economic cost of diagnosing mitochondrial diseases was significantly correlated with the year of disease onset (*P* < 0.001). The median annual economic cost for treatment and symptom management after diagnosis was $12,292.79, with direct medical and non-medical costs of $10,887.53 and $1,360.44, respectively, and an indirect cost of $5,442.21. Healthcare insurance covered only 15.16% of direct medical expenses. No significant differences were observed between the subgroups after diagnosis and the annual economic costs of treatment or symptom management.

**Conclusion:**

The study findings indicated that the economic burden of both the diagnosis and treatment of patients with mitochondrial diseases was substantial. Increased emphasis should be placed on primary and secondary prevention strategies to further reduce the overall economic burden of rare genetic diseases, such as mitochondrial diseases.

**Supplementary Information:**

The online version contains supplementary material available at 10.1186/s13023-025-03708-1.

## Background

Mitochondrial diseases (MDs) are a group of energy metabolism disorders characterised by mitochondrial protein dysfunction due to genetic defects. The incidence of MDs among live births is approximately 1.6/5,000 [1]. In childhood, MDs primarily manifest as Leigh syndrome (LS) or mitochondrial encephalomyopathy, lactic acidosis, and stroke-like episodes (MELAS), which seriously affect the patient’s neurological and motor functions and are major causes of death and disability in children.

The diagnosis of MDs is challenging because of the wide variability in the signs and symptoms present in patients. Moreover, diagnosis is further complicated by a lack of sensitive and specific biomarkers, as well as the cost and invasiveness of some tests, such as muscle biopsy, which has historically been used for diagnosis. The gold standard for diagnosing MDs is genetic testing; however, this is an expensive process that is often not covered by health plans, and > 50% of patients with MDs cannot be diagnosed after whole exome sequencing (WES) [2]. MDs are chronic diseases involving multiple systems. Due to the lack of effective treatment options, management is primarily based on multidisciplinary symptomatic treatment and multi-vitamin ‘cocktail therapy’ [3, 4]. Therefore, the diagnosis and treatment of MDs impose a significant economic burden on families and society. As early as 2012, a cross-sectional study conducted in the United States on the hospitalisation costs of 3200 children with MDs [5] showed that the per capita annual direct medical expenses related to MDs were $25,000.00, with a median of $9,037.00, indicating the considerable economic burden of MDs.

Since 2012, with the widespread application of next-generation sequencing (NGS) technology, the number of confirmed patients with MDs has rapidly increased, and the demand for management and treatment after diagnosis has also expanded. Although the cost of WES has decreased from $3,000.00 in 2012 to $1,000.00 in 2023, the effect of this reduction on the economic burden of patients remains unclear. Moreover, it remains unclear whether the time to diagnosis can be shortened and whether treatment options have progressed. In addition, for rare diseases such as MDs, establishing an effective medical security system would likely help reduce the economic burden on patients and families and protect their rights and interests. Universal healthcare has been widely promoted in China; however, with varying medical insurance policies across China’s vast regions, the extent of medical insurance coverage for MDs and whether such coverage can improve the economic burden on patients remains unclear. To provide effective improvement measures, it is crucial to understand the factors that influence the economic burden. However, to the best of our knowledge, no study has evaluated the economic burden or factors influencing MDs in China.

Therefore, this study aimed to investigate the economic cost of patients with genetically confirmed MDs at Beijing Children’s Hospital, affiliated with Capital Medical University, a national centre specialising in the diagnosis of paediatric MDs. The study findings may help address the current lack of data on the medical and economic burden of MDs in China and provide a foundation for further guidance in clinical decision-making and efforts to reduce this burden.

## Methods

### Research design

In this single-centre, cross-sectional study, a randomly administered online questionnaire was sent to patients who had been diagnosed with MDs through genetic testing and who had been registered with the Chinese Mitochondrial Disease Network (MitoC-NET, ChiCTR1900028101) between January 2012 and January 2022. Given the challenges in diagnosing and treating MDs, this survey was divided into two parts: the total economic cost of diagnosing MDs and the annual costs of treatment and symptom management after diagnosis. As most of the patients were underaged, family members completed the questionnaire. This study was approved by the Ethics Review Committee of Beijing Children’s Hospital, affiliated with Capital Medical University ([2022]-E-121-Y) and was conducted in accordance with the ethical guidelines of the Declaration of Helsinki.

### Design of the questionnaire survey

The questionnaire mainly covered the following parts (see Supplementary Material): (i) demographic and societal information (e.g., name, gender, age of onset, year of onset, parental education, geographic region, living area, annual household income, and healthcare insurance type); (ii) clinical information (e.g., clinical phenotype, siblings with similar diseases); and (iii) economic burden (e.g., direct medical costs, which refer to various costs incurred within or outside hospitals for the diagnosis, treatment, and rehabilitation of MDs; and direct non-medical costs, which refer to transportation, accommodation, catering, nursing, special education, and psychological counselling costs incurred during the diagnosis, treatment, and management of MDs; and indirect costs, which refer to the income lost by caregivers having sought medical treatment or in caring for patients). The healthcare insurance ratio refers to the proportion of reimbursements to direct medical costs. The exchange rate used was 1 USD = 7.1668 RMB on October 25, 2022.

### Process and quality control

Neurologists and patient families were invited to complete the initial questionnaire. Based on their feedback, the content of the questionnaire was adjusted to design the final version, which was published online. All participants received a standard consultation and training program prior to completing the questionnaire. A neurologist explained each item in the questionnaire to the participants to ensure that they understood the meaning of the questions and completed them correctly. The research team then conducted segmented evaluations of the reliability of the collected data. The questionnaire included a multilevel quality control procedure in which participants performed self-evaluation, and the quality control staff then reviewed the results again within two days after the survey. If inaccurate data were found, the neurologists verified the data through phone interviews, and only eligible patients were included in this study.

### Statistical analyses

Statistical analyses were performed using SPSS 25.0 software (IBM Corp., Armonk, NY, USA) to describe patient demographic data, disease-related information, and economic costs. Normally distributed data are denoted as mean ± standard deviation. Non-normally distributed data are denoted as the mean, median, and 25th and 75th quantiles. When analysing factors that affect economic costs, two independent samples were compared using a Mann–Whitney *U* test, and more than two groups were compared using a Kruskal–Wallis test. Additionally, the generalised linear model gamma conjugate (A1) was used for multivariate analysis, and a natural logarithm transformation was applied to expenditure data with high dispersion. *P*-values < 0.05 were considered statistically significant.

## Results

From January 2012 to January 2022, 684 genetically confirmed MD cases were registered in MitoC-NET, with 145 completed survey questionnaires initially obtained, after which 43 patients with incomplete data were excluded. In total, 102 patients were included in this study (boys, *n* = 55; girls, *n* = 47; mean onset age, 6.71 ± 6.62 years; Table 1). Ten patients reported disease onset before 2012, 28 patients reported onset between 2012 and 2016, and 68 patients reported onset after 2016. These patients had various clinical phenotypes, most frequently MELAS (*n* = 49), followed by LS (*n* = 23), mitochondrial myopathy (*n* = 15), and myoclonic epilepsy and ragged-red fibres (*n* = 15), with 11 (10.78%) patients also having siblings with an MD. From a genetic perspective, the MT - TL1 m.3243 A > G mutation was the most common (total, *n* = 46 cases), and this mutation triggered the MELAS phenotype in all instances.

**Table 1:** Characteristics of the patient sample (*n* = 102)

Variables	*n* (%)
**Gender**	
Male	55 (53.92)
Female	47 (46.08)
**Age of onset (years)**	6.71 ± 6.62
< 1	23 (22.54)
1–3	13 (12.75)
4–6	17 (16.67)
7–12	32 (31.37)
13–18	17 (16.67)
**Year of onset (years)**	
Before 2012	10 (9.80)
2012–2016	28 (27.45)
After 2016	64 (62.75)
**Mitochondrial syndromes**	
MELAS syndrome	49 (48.04)
Leigh syndrome	25 (24.51)
MM	15 (17.01)
MERRF	4 (3.92)
Others	9 (8.82)
**Siblings with similar diseases**	
Yes	11(10.78)
No	91(89.22)
**Geographic region**	
Eastern region	51 (50.00)
Central region	34 (33.33)
Western region	17 (16.67)
**Living area**	
City residents	45(44.12)
Urban residents	24(23.53)
Rural residents	33(32.35)
**Healthcare insurance type**	
Urban medical insurance	41 (40.19)
New rural cooperative medical care	39 (38.24)
Other type of medical insurance	17 (16.67)
Self-paying	5 (4.80)
**Parental education**	
Junior high school or below	31 (30.39)
Senior High school	15 (14.71)
Undergraduate or over	56 (54.90)
**Annual household income($)**	
< 10,000$	58 (56.86)
10,000–20,000$	31 (30.39)
> 20,000$	13 (12.75)

MELAS, mitochondrial encephalomyopathy, lactic acidosis, and stroke-like episodes; MM, mitochondrial myopathy; MERRF, myoclonic epilepsy and ragged-red fibres

**Fig. 1:**
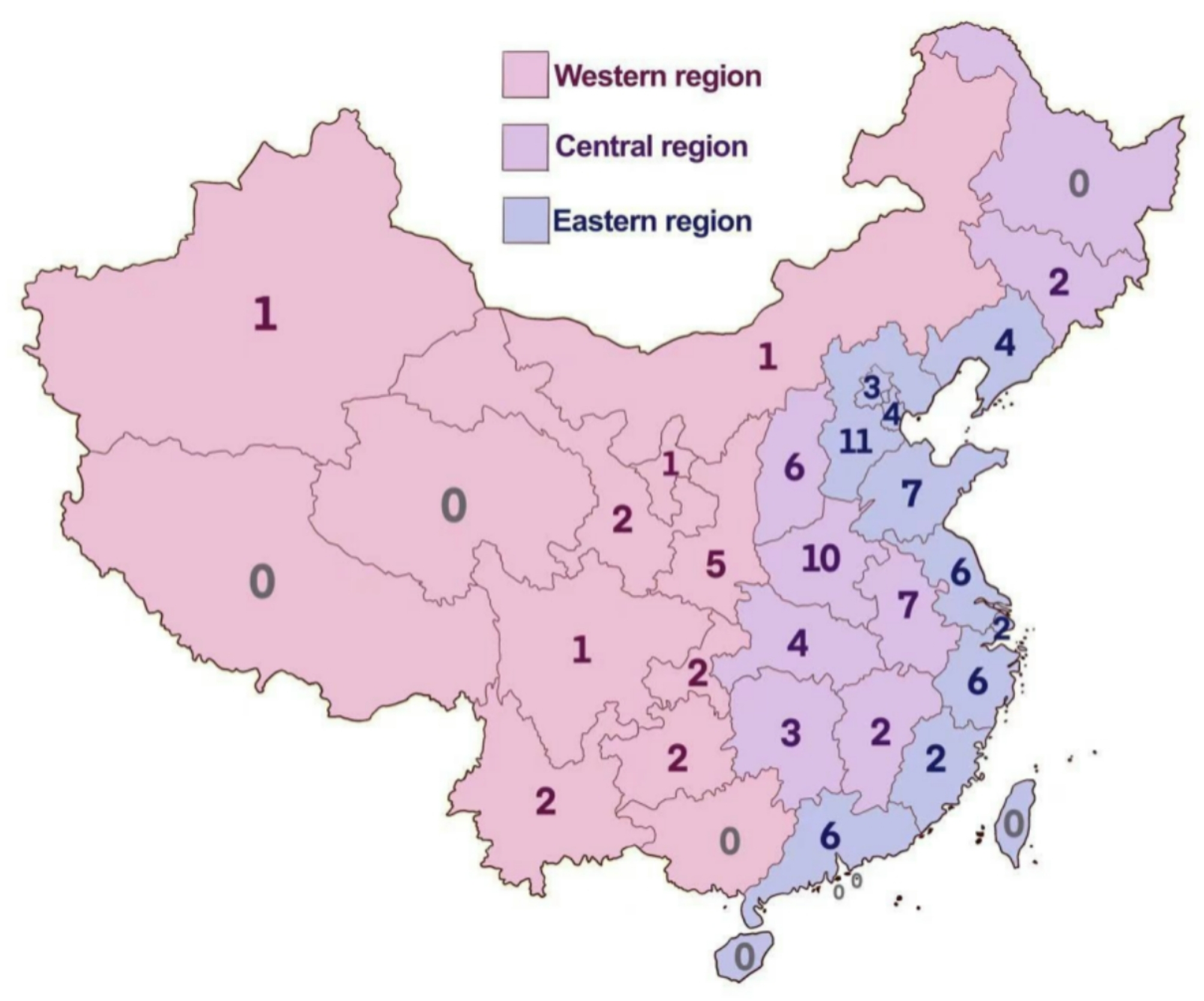
Distribution of the study patients with mitochondrial diseases

The 102 study patients came from different regions of China (Fig. 1), including the western (*n* = 17), eastern (*n* = 51), and central (*n* = 34) regions. Among them, 45 patients were inner-city residents (44.12%), 24 were urban residents (23.53%), and 33 were rural residents (32.35%). The median annual family income was $9,867.56. A total of 97 (95.2%) patients were covered by various healthcare insurance programmes, and 56 (54.90%) had an undergraduate degree or higher (Table 1).

The mean total direct economic cost incurred in diagnosing MDs was $13,751.89 per case, with a median of $8,520.19, whereas the mean annual cost of treatment and symptom management after diagnosis was $14,849.95 per case, with a median of $12,292.79 (Table 2). Both costs were mainly direct medical costs, accounting for 82.20% and 87.66% of the total costs. The highest proportion of direct medical costs for diagnosis was mainly related to examination costs (62.05%), whereas following a diagnosis of MD, treatment costs were the highest priority (81.96%), with examination costs decreasing to 6.50%. In terms of healthcare insurance coverage, direct medical costs before and after diagnosis accounted for 27.29% and 15.16% of the costs, respectively. The mean direct non-medical costs before and after a diagnosis of an MD were $2,365.47 and $1,831.96, respectively, with median costs of $2,092.98 and $1,360.44, respectively. Transportation costs accounted for the highest proportion of both direct medical and non-medical costs (36.71% and 25.14%, respectively). In addition, indirect costs also posed an important economic burden, with mean indirect costs of $6,532.69 and $7,072.94 per case before and after diagnosis and median costs of $3,162.93 and $5,442.21, respectively (Table 2).

**Table 2:** Economic cost composition of costs related to mitochondrial disease

Subjects	Total cost required for diagnosis				Annual costs for management and treatment after diagnosis				
	Percent (%)	Mean	Median	q25	q75	Percent (%)	Mean	Median	q25 q75
**Total direct cost($)**	100.00	13,751.89	8,520.19	5,501.06	19,058.8	100.00	14,849.95	12,292.79	7,400.44 18,319.72
**Direct medical costs($**)^a^	**82.80**	11,386.42	6,769.06	3,850.22	15,123.99	**87.66**	13,017.99	10,887.53	6,490.87 16,471.79
registration costs^b^	1.43	162.75	78.48	26.16	220.65	0.80	104.47	57.56	23.55 109.89
Treatment costs^b^	36.52	4,158.56	2,598.93	1,491.33	4,927.51	81.96	10,669.61	6,540.94	4,657.15 12,366.74
Examination costs^b^	62.05	7,065.27	4,447.96	1,691.97	8,614.84	6.50	846.78	575.92	331.51 1046.67
Rehabilitation costs^b^	-	-	-	-	-	10.73	1,397.13	0.00	0.00 3488.85
**Direct non-medical costs($)** ^a^	**17.20**	2,365.47	2,092.98	1,081.38	3,383.66	**12.34**	1,831.96	1,360.44	767.43 1970.89
Transportation costs^b^	36.71	868.42	697.72	418.63	1,534.98	25.14	460.63	348.86	209.32 627.95
Accommodation costs^b^	26.43	625.25	418.62	139.54	976.78	16.13	295.50	244.20	174.43 383.74
Catering costs^b^	17.33	409.83	348.85	209.31	627.93	14.03	257.07	244.20	174.43 363.21
Nursing costs^b^	19.53	461.99	209.31	69.77	690.70	21.89	401.02	209.31	69.77 627.93
Special education costs^b^	-	-	-	-	-	22.36	409.69	0.00	0.00 0.00
Psychological counselling costs^b^	-	-	-	-	-	0.44	8.13	0.00	0.00 0.00
**Indirect cost($)**	-	6,532.69	3,162.93	1,011.69	8,610.68	-	7,002.94	5,442.21	3,676.68 12,367.08
**Healthcare insurance($)** ^**c**^	**27.29**	3,107.66	2,523.20	1,413.75	5,344.50	**15.16**	1,973.42	1,545.14	1,001.25 4,464.95

Note: ^a^represents the percentage of the total mean direct cost, ^b^represents the percentage of the mean direct medical/nonmedical costs, and ^c^represents the percentage of the mean direct medical cost. ‘-’ Indicates that this part has not been investigated

To further understand the factors influencing economic costs, we summarised the economic costs of the different subgroups and conducted univariate analysis based on sex, age at onset, year of onset, clinical phenotype, siblings with similar diseases, geographic region, living area, parental education, and annual household income. Significant differences were observed between the direct economic costs before the diagnosis of an MD and the year of onset (Table 3, *P* < 0.001), which was also confirmed using multivariate analysis (Table 4). The median direct economic cost for diagnosis before 2012 was $27,789.62, which decreased to $14,792.17 between 2012 and 2016 and further dropped to $6,366.34 after 2017. After diagnosis, no statistically significant differences were observed between the subgroups (Tables 3 and 4).

**Table 3:** Overall and subgroup analysis of medical and non-medical cost for diagnosis and treatment of per patient with mitochondrial disease

Variables		Total direct cost required for diagnosis					annual direct cost for management and treatment after diagnosis				
	Medical cost	Non-medical cost	Total cost	Statistics		*P*	Medical cost	Non-medical cost	Total cost	Statistics	*P*
**Total cost($)**	6,769.06	2,092.98	8,520.19				10,887.53	1,360.44	12,292.79		
**Gender**											
Male	7,700.44	2,232.52	8,853.32	-1.014		0.311	12,189.89	1,395.32	13,407.31	-1.067	0.286
Female	7,736.52	2,092.98	8,403.92				11,493.43	1,255.79	12,470.15		
**Age of onset (years)**											
< 1	4,962.12	1,953.45	7,521.39	8.826		0.066	8,460.89	1,465.09	10,641.08	9.21	0.056
1–3	7,902.76	2,930.18	8,853.32				8,699.29	1,255.79	9,815.55		
4–6	7,147.54	1,813.92	9,659.12				12,213.71	1,011.61	14,016.02		
7–12	7,934.15	2,092.98	9,863.19				11,307.35	1,116.26	13,281.73		
13–18	6,661.96	2,372.05	7,708.39				13,332.31	1,604.62	15,127.04		
**Year of onset (years)**											
Before 2012	23,185.03	3,209.24	27,789.62	35.638		**< 0.001**	13,006.15	1,674.39	14,680.54	1.306	0.52
2012–2016	12,052.1	2,790.65	14,792.17				10,975.96	1,395.32	13,407.31		
After 2016	4,960.37	1,604.62	6,366.34				10,182.37	1,255.79	11,159.09		
**Mitochondrial syndromes**											
MELAS syndrome	7,934.15	2,232.52	9,400.39				12,779.41	1,325.56	13,975.9		
Leigh syndrome	5,258.62	2,372.05	7,613.23	4.061		0.398	10,010.93	1,465.09	11,475.99	2.168	0.705
MM	5,628.38	1,953.45	7,163.24				11,000.38	1,465.09	11,558.51		
MERRF	7,443.12	2,651.11	8,094.23				9,601.57	906.96	10,321.94		
Others	7,897.53	1,534.86	8,853.32				10,431.78	767.43	10,641.08		
**Siblings with similar diseases**											
Yes	6,141.76	1,360.44	8,327.79	1.514		0.133	9,693.46	1,151.14	10,426.01	0.354	0.723
No	7,048.12	2,162.75	8,929.19				10,988.17	1,360.44	12,507.33		
**Geographic region**											
Eastern region	5,935.35	2,092.98	7,665.56	2.347		0.309	10,975.96	1,116.26	12,144.54	1.343	0.511
Central region	6,496.97	2,092.98	8,926.58				12,064.31	1,465.09	13,477.07		
Western region	6,769.34	2,790.65	8,443.39				9,493.43	1,255.79	11,330.62		
**Living area**											
City residents	7,142.25	2,511.58	9,729.43	5.012		0.082	10,615.46	1,534.86	12,014.27	3.502	0.174
Urban residents	4,840.90	2,023.22	6,721.97				8,399.84	1,220.91	9,498.66		
Rural residents	6,294.65	1,813.92	8,003.92				9,475.99	1,116.26	10,330.62		
**Parental education**											
Junior high school or below	7,934.15	1,953.45	10,909.68	4.534		0.104	13,006.15	1,116.26	13,975.93	1.003	0.606
Senior High school	8,843.43	2,790.65	10,331.71				13,332.31	1,255.79	14,657.87		
Undergraduate or over	7,483.79	2,023.22	9,328.93				11,847.49	1,465.09	13,555.62		
**Annual household income ($)**											
< 10,000	7,461.49	2,092.98	9,334.71	0.638		0.727	11,054.4	1360.44	12,507.33	2.243	0.326
10,000–20,000	6,073.14	2,023.22	8,707.41				9,209.13	1220.91	10,014.93		
> 20,000	6,113.26	2,372.05	9,270.18				10,779.41	1499.97	11,710.79		

**Table 4:** Multivariate analysis of overall direct economic cost for diagnosis and treatment of per patient with mitochondrial disease

Variables	Total direct cost required for diagnosis		annual direct cost after diagnosis	
	Estimate(95%CI)	*P*	Estimate (95%CI)	*P*
**Intercept**	9.452 (8.872 ~ 10.032)	0.000	9.625 (9.339 ~ 9.912)	0.000
**Age of onset (years)(Ref = < 1)**				
1–3	0.265 (-0.133 ~ 0.663)	0.191	-0.127 (0.500 ~ 0.246)	0.505
4–6	0.251 (-0.182 ~ 0.684)	0.256	0.264 (-0.137 ~ 0.665)	0.197
4–12	0.626 (0.248 ~ 1.005)	0.001	0.118 (-0.229 ~ 0.465)	0.504
13–18	0.461 (-0.01 ~ 0.932)	0.055	0.058 (-0.374 ~ 0.489)	0.793
**Year of onset (years) (Ref = Before 2012)**				
2012–2016	-0.718 (-1.161 ~ 0.275)	**0.001**	-	-
After 2016	-1.233 (-1.612 ~ 0.853)	**< 0.001**	-	-
**Siblings with similar diseases (Ref = Yes)**				
No	0.574 (0.105 ~ 1.043)	0.163	-	-
**Living area (Ref = City residents)**				
Urban residents	0.165 (-0.187 ~ 0.518)	0.359	-0.117 (-0.436 ~ 0.202)	0.473
Rural residents	-0.104 (-0.445 ~ 0.237)	0.549	-0.176 (-0.468 ~ 0.116)	0.237
**Parental education (Ref = Junior high school or below)**				
Senior High school	0.216 (-0.25 ~ 0.682)	0.363	-	-
Undergraduate or over	-0.073 (-0.431 ~ 0.284)	0.688	-	-

95% CI, 95% confidence interval; ‘-’ indicates not included in the multivariate analysis

## Discussion

To the best of our knowledge, this is the first study to provide a comprehensive overview of the economic costs of MDs in paediatric patients in China. MDs are rare and present challenges in terms of both diagnosis and treatment. Delays in diagnosis and treatment are common owing to limited public awareness. Moreover, the high rates of disability and mortality associated with MDs exacerbate the economic burden on affected families. However, studies on this topic are lacking. Using questionnaire data, we aimed to raise awareness about the existence and serious economic burden of MDs and to facilitate a multidisciplinary approach to address them.

Overall, healthcare costs for those diagnosed with MDs were high for both diagnosis and treatment. The mean direct medical cost for diagnosing MDs was US$11,386.42 (median, $6,769.06) per case, while the mean direct medical cost for treatment and symptom management after diagnosis was $13,017.99 (median, $10,887.53) per case. In the United States, per-person direct medical expenses related to MDs alone were reported to be as high as $25,000 per year [5]. In Canada, the mean cost incurred in the 12 months prior to hospitalisation was reported to be $24,023 (median, $9,839), and the mean cost incurred in the 12 months post-discharge was $33,545 (median, $11,445) [6]. The direct medical expenses for patients with MDs in China were lower than those for patients in the United States and Canada; however, the median annual income of patients in this group was only $9,867.56, with > 50% of the households incurring direct medical burdens that exceeded their total annual income. The overall healthcare insurance coverage rate for this patient group reached 95.2%; however, the average reimbursement amount per patient in terms of direct medical costs is extremely low, at only 27.29% before diagnosis and decreasing to 15.16% after diagnosis. This is mainly because the initial healthcare insurance reimbursement rate in China for rare diseases such as MDs is not high. Moreover, most patients concurrently seek medical treatment elsewhere, which further reduces the reimbursement rate. In addition, some tests, such as gene sequencing and metabolic screening of blood and urine, along with certain treatments not covered by healthcare insurance, lead to increased economic burdens on patients’ families.

The mean direct non-medical cost incurred for diagnosing MDs accounted for 17.20% of the direct economic cost, whereas the mean direct non-medical cost for treatment and symptom management after diagnosis decreased to 12.34%. With increased outpatient follow-ups and a reduced number of unnecessary hospitalisations after an MD diagnosis, transportation and accommodation costs have decreased. However, some local hospitals are unable to manage acute MD-related complications. Establishing regional medical centres and expanding Internet-based medical services could further reduce this burden.

The indirect costs for patients with MD are also high, as MDs primarily affect the central nervous system and muscles, are chronic and progressive, and have high disability rates. Most children require long-term treatment and care, and their caregivers are unable to engage in normal work activities for many years. In our survey, most patients were cared for by their parents, and some families had multiple affected members. To reduce the economic burden on families, it is crucial to effectively control this condition.

The cost of examination was the highest among the various economic costs before diagnosis. Genomics has been widely applied in diagnosis; however, it is expensive and not covered by healthcare. Moreover, the incidence of MDs in children is highly heterogeneous and involves multiple systems, requiring extensive examinations that increase the economic cost of diagnosis. Our findings indicate that with progress in genetic testing technology, the time taken to diagnose MDs has significantly shortened, helping to significantly reduce the cost of medical treatment for patients. These cost reductions include avoiding expenses incurred from unnecessary tests, evaluations, and treatments during confirmation of the diagnosis, while also minimizing costs related to repeated patient visits, such as transportation and accommodation costs. Prior to 2012, our centre primarily used PCR-RFLP or Sanger sequencing methods for detection. From 2012 to 2016, we primarily used panel sequencing. After 2016, WES became the preferred method. The diagnostic time for MDs has significantly shortened, decreasing from 35 months before 2012 to 17 months before 2016, and further decreasing to 3 months after 2016. Diagnostic costs have also continuously decreased. Starting from $27,789.62 per case initially, this cost first decreased to $24,322.15 and finally to $6,366.34, with an overall reduction of 77.09%. Additionally, no correlation was observed between the economic cost of diagnosing MDs and the geographical distribution of patients, parental education, or similar factors. These data indicate that genetic testing has become widely available in China and that doctors at all levels have greatly improved their understanding of MDs or hereditary diseases.

Among the annual economic costs after diagnosis, treatment costs accounted for the highest proportion of the total economic cost, rising from 20.50% before diagnosis to 48.82% after diagnosis, while the examination costs decreased from 34.83% before diagnosis to 3.87% after diagnosis. In this study, 94 of 102 patients (92.16%) were treated with ‘cocktail therapies’ such as idebenone, L-carnitine, coenzyme Q10, vitamins B and E, and nicotinamide mononucleotide. More than 50% of the patients were taking > 5 drugs (up to 13 at most) for long-term use in large doses, with most of the drugs comprising health products not covered by healthcare insurance, resulting in a significant increase in treatment costs. This phenomenon is due to China’s unique culture, where even if medication is ineffective, some people will persist in taking it, and some families may even seek traditional Chinese medicine treatment. Despite the number of clinical trials of MD drugs is gradually increasing, the rarity and complexity of each type of MD, along with the lack of natural history research, pose significant challenges to these trials, emphasising the need for global, multicentre research. Genetic therapies targeting mtDNA include selective elimination of mutant mtDNA using restriction enzymes delivered as mitoTALENs [7] or replacing a mitochondrial protein by expressing it in the nucleus, an approach currently under clinical trial for Leber hereditary optic neuropathy [8]. Adeno-associated virus-mediated gene therapy directed at nuclear-encoded mitochondrial defects has been reported in a number of mouse models [9, 10] but has yet to be translated to human MDs. If gene therapy for MDs becomes applicable in clinical practice in the future, this type of treatment is likely to lead to a significant increase in direct medical costs. However, if the disease symptoms are alleviated after patients have received gene therapy, the transportation costs incurred owing to frequent medical visits and hospitalization, along with the accompanying costs to family members, may be reduced.

To formulate sustainable health policies for rare diseases such as MDs and to develop appropriate public health policies, attention needs to be paid to patient costs and the broader socioeconomic effects. To address these issues, it is necessary to identify the key issues. In terms of primary prevention, it is crucial to conduct comprehensive mitochondrial gene screening for individuals with a relevant family history. Through identifying individuals carrying mitochondrial pathogenic mutation genes at an early stage, genetic counselling can be provided, and guidance can be provided in terms of making fertility decisions, thus blocking the inheritance of the disease to the next generation at the source. In the field of secondary prevention, while MDs currently remain incurable, early diagnosis and treatment can maximise the physiological functions, prevent exacerbation of medical costs, prolong the survival time, and lead to improvements in quality of life for some patients. For healthcare institutions, the most critical aspect currently is the overall improvement in diagnosis and treatment. Improvements in diagnostic efficiency can reduce the time and economic costs required for diagnosis. However, to improve the treatment effect, in addition to increasing research and development of orphan drugs, it is necessary to apply a multidisciplinary approach involving disciplines such as rehabilitation, nursing, psychology, and special education. In our study, few families chose to provide rehabilitation training, psychological treatment, or special education to the patients. The reasons for this may lie in the severity of the disease itself as well as parents’ lack of awareness or shortage of resources in relevant domestic institutions.

Despite being the first economic cost survey of MDs in children in China, this study had several limitations. The study sample size was small, the clinical phenotypes of MDs were numerous, and the study did not fully investigate all types of economic burdens. Moreover, the number of cases in each region was limited, which may have affected the representativeness of the findings. Our research was conducted using a survey questionnaire, and retrospective data concerning diagnosis- and treatment-related costs were collected, which may have introduced data errors.

## Conclusion

The economic burden of diagnosing and managing MDs is substantial. To further reduce the overall economic burden of rare genetic diseases such as MDs, more efforts should be directed toward strategies for primary and secondary prevention.

## Electronic supplementary material

Below is the link to the electronic supplementary material.

Supplementary Material 1

Supplementary Material 2

## Data Availability

The data that support the findings of this study are available from the corresponding author upon reasonable request.

## Abbreviations

MD: Mitochondrial diseases
LS: Leigh syndrome
MELAS: Mitochondrial encephalomyopathy, lactic acidosis, and stroke-like episodes
MM: Mitochondrial myopathy
MERRF: Myoclonic epilepsy and ragged-red fibres
WES: Whole exome sequencing
NGS: Next-generation sequencing
