# Supplementary material for: Preliminary investigation on the economic cost of mitochondrial disease in Chinese children
Source: Orphanet J Rare Dis. 2025 Apr 10;20:172. doi: 10.1186/s13023-025-03708-1 (PMC11987409; doi:10.1186/s13023-025-03708-1)
Supplement: Supplementary file 1 — Supplementary Material 1 [file 13023_2025_3708_MOESM1_ESM.doc]

**Questionnaire on the Economic Burden of Mitochondrial Diseases in Chinese Children**

Dear parents,

To gain an in-depth understanding of the practical needs of families with children diagnosed with mitochondrial disease in terms of family economy and social security, we have carefully designed this questionnaire. This survey is of considerable significance, mainly because it can help us to understand the economic burden faced by patients with mitochondrial disease and their families and because it can provide strong support for the formulation of relevant social security payments, welfare policies, and social support systems for mitochondrial diseases. The content of the questionnaire is divided into three parts. Part 1 comprises basic information. Part 2 focuses on the economic cost situation before the genetic diagnosis. Part 3 refers to the economic cost situation after diagnosis.

We thank you very much for taking the time to participate in this questionnaire survey. Every aspect of your feedback will help us to have a clearer understanding of the social situation in relation to this disease group and make key contributions to improving the social security system for patients with mitochondrial disease.

**Part 1. Basic information. Please respond in as much detail as possible according to your current situation.**

1. Patient's name:

2. Patient's sex:

3. Patient's date of birth:

4. Region where the patient is from:

5.Patient's current residence area:

6. The highest education level of both of the patient's parents:

7. The patient’s age at disease onset:

8. The patient's clinical diagnosis:

9. The age at which the patient received a genetic diagnosis:

10. The pathogenic gene that caused the patient's disease:

11. Is there is a similar medical history among the patient's siblings:

12. The type of patient healthcare insurance:

13. Your family's annual total income:

**Part 2. This part refers to medical treatment expenses before the genetic diagnosis, including your expenses in outpatient and emergency departments, hospitalization, and other aspects. Please complete these questions as accurately as possible according to your current situation.**

14. How many times did the patient visit the outpatient and emergency department in total before the diagnosis?

15. What was the average cost of each visit to the outpatient and emergency department by the patient before the diagnosis?

16. How much did you spend on registration as part of outpatient and emergency department visit expenses?

17. How much did you spend on examinations as part of outpatient and emergency department visit expenses?

18. How much did you spend purchasing medications as part of outpatient and emergency department visit expenses?

19. How much was reimbursed after the outpatient and emergency department visits?

20. What was your total expenditure on medications purchased on your own (including those purchased at pharmacies, on online pharmacies, and via overseas purchasing agents, etc.) before the diagnosis?

21. Among the above-mentioned various expenses, which ones do you consider to be relatively more expensive?

22. Can these expenses be reimbursed? If so, what is the reimbursement rate?

23. How many times in total was the patient hospitalized owing to this disease in total before the diagnosis?

24. What was the average cost of each hospitalization of the patient before the diagnosis?

25. What was the average cost of the examinations undertaken during hospitalization?

26. How much did you spend on medication treatment during hospitalization?

27. How much of the hospitalization expenses were reimbursed?

28. Among the hospitalization-related expenses, which ones do you think were relatively more expensive?

29. Can these hospitalization expense be reimbursed? What is the reimbursement rate?

30. How much was the total transportation cost incurred as a consequence of seeking medical treatment before the diagnosis?

31. How much was the total accommodation cost incurred due to seeking medical treatment before the diagnosis?

32. How much was the total catering cost incurred due to seeking medical treatment before the diagnosis?

33. What was your total cost incurred concerning the requirement for nursing consumables (including nursing pads, disposable diapers, wheelchairs, nasogastric feeding nursing consumables, etc.) required due to the disease before the diagnosis?

34. How much was the total loss of wages owing to patient medical visits or the family members' care for the patient before the diagnosis?

**Part 3: This part refers to the average annual expenditure after the patient was genetically diagnosed with mitochondrial disease. Please fill in as accurately as possible according to the actual situation.**

35. How many times has the patient visited the outpatient and emergency department on average each year after being diagnosed?

36. What is the average cost of each visit to the outpatient and emergency department for the patient after diagnosis?

37. Among these costs, how much is the cost for registration during the outpatient and emergency department visits?

38. Among these costs, how much is the cost for examinations during the outpatient and emergency department visits?

39. Among these costs, how much is the cost for purchasing medications during the outpatient and emergency department visits?

40. What is the final reimbursed amount of the outpatient and emergency department expenses?

41. After the genetic diagnosis, what has been your average annual expenditure on various medications purchased externally (including those purchased from physical pharmacies, online pharmacies, and obtained through overseas purchasing agents, etc.)?

42. Among all the above-mentioned expenditure items, which ones do you think are relatively the most expensive?

43. Could these expenses be included in the reimbursement scope?

44. How many times has the patient been hospitalized on average each year due to the disease after diagnosis?

45. What is the specific average cost of each hospitalization for the patient after diagnosis?

46. Among these costs, how much is the cost for examinations during hospitalization?

47. Among these costs, how much is the cost for medication treatment during hospitalization?

48. What is the actual reimbursed amount of the hospitalization expenses?

49. What is the average cost for the patient's rehabilitation treatment each year?

50. Among the hospitalization-related expenses and the rehabilitation treatment expenses, which ones do you think are relatively more expensive?

51. Can these expenses be reimbursed?

52. How much is the total transportation cost incurred due to seeking medical treatment each year after diagnosis?

53. How much is the total accommodation cost incurred due to seeking medical treatment each year after diagnosis?

54. What has been the total annual catering cost incurred for seeking medical treatment following the diagnosis?

55. What has been the total annual cost of nursing consumables in relation to the patient's disease following the genetic diagnosis (covering nursing pads, disposable diapers, consumables for changing dressings of tracheal intubation, sputum suction catheters, wheelchairs, consumables related to nasogastric feeding nursing, etc.)?

56. Is the patient currently receiving special education (such as receiving education in specialized kindergartens, special education schools, or special education institutions)?

57. How much of your expenses are incurred investing in the patient's special education annually?

58. Do family members require psychological counselling because of the patient's illness?

59. How much is spent on psychological counselling each year?

60. What is your (or your family members’) total annual loss of income resulting from having to care for and accompany the patient to seek medical treatment?

61. Are you satisfied with the patient’s current diagnosis and treatment outcomes?

62. Please provide your phone number so that we can contact you later.
