# Supplementary material for: Preliminary investigation on the economic cost of mitochondrial disease in Chinese children
Source: Orphanet J Rare Dis. 2025 Apr 10;20:172. doi: 10.1186/s13023-025-03708-1 (PMC11987409; doi:10.1186/s13023-025-03708-1)
Supplement: Supplementary file 2 — Supplementary Material 2 [file 13023_2025_3708_MOESM2_ESM.docx]

Dear Dr. Fang,

Your manuscript 'Preliminary investigation on the economic cost of mitochondrial disease in Chinese children' (OJRD-D-24-01364) has been assessed by our reviewers. Although it is of interest, we are unable to consider it for publication in its current form. The reviewers have raised a number of points which we believe would improve the manuscript and may allow a revised version to be published in Orphanet Journal of Rare Diseases.

Dear Editor,

Thank you for your consideration and coordination with the reviewers in relation to our manuscript. We also thank both reviewers for their valuable comments and suggestions, which have helped us to strengthen our study. We have addressed all of the concerns by adding new data and revising the text in response to the reviewers’ requests. Our changes to the manuscript are highlighted in yellow. Our point-by-point responses to the comments raised by the reviewers are as follows.

Reviewer reports:

**Reviewer #1**: Thank you to the authors for allowing me the opportunity to review their scientific work, which analyzes the direct and indirect costs of suffering from mitochondrial disease in China. For a better understanding of the article, the following changes are suggested:

- Line 39: primary and secondary prevention strategies: It is suggested to detail possible strategies in primary and secondary prevention, taking into account the mitochondrial inheritance of some of these diseases.

**Response:** Thank you for your extremely important suggestion. We have added further content on **page 14, lines 261–269** of the Discussion section.

- Line 48: "In childhood, MDs primarily manifest as Leigh syndrome (LS) or mitochondrial encephalomyopathy, lactic acidosis and stroke-like episodes …… ". It is suggested to define diseases, not the symptoms associated with the diseases, it would be enough to say: MELAS or Leigh syndrome.

**Response:** Thank you for this suggestion. I fully understand what you mean. However, the abbreviation ‘MELAS’ is defined as ‘mitochondrial encephalomyopathy, lactic acidosis, and stroke-like episodes.’ In accordance with the *Journal* guidelines, we have defined the abbreviation ‘MELAS’ on first use in the main text on **lines 47–50**.

- Line 55: Could you explain your profitability with WES if you have the data and if you have found secondary findings of other mutations in accessible genes? And since you know a group of mitochondrial diseases in your area, why don't you first analyze an NGS panel of genes before the whole exome? This could make the cost of the study less expensive.

**Response:** We sincerely appreciate your insightful questions. Unfortunately, through analyzing WES data, we did not identify any secondary findings related to other mutations among the accessible genes. However, we have identified some mitochondrial co-pathogenic mutations. For instance, the co-existence of a heterozygous mutation in the nuclear gene EARS2 and the mitochondrial gene MT - TE m.14674T>C implies a potential co-pathogenic mechanism (unpublished data).

Regarding the protocol of performing panel sequencing prior to WES, for certain patients with distinctly suspected genes (with MELAS as a prime example), the most prevalent mutation currently is m.3243A>G. For these patients, we typically opt for panel sequencing as the initial approach. Conversely, for a substantial number of other patients lacking any suspected genes, we tend to directly employ WES more frequently. Through utilizing WES, we are able to comprehensively and exhaustively capture all potential genetic variations, encompassing both those that are already known and those yet to be discovered. Admittedly, in comparison with panel sequencing, WES might lead to an increase in direct medical costs for some individuals. However, when viewed from the perspective of the entire patient cohort, it actually reduces the economic burden for most patients. Our data strongly validate this trend. For detailed information, kindly refer to **page 12, lines 227–231** of the revised manuscript.

- Lines 216 y 216 y 218 : "cost of diagnosis. Our findings indicate that with progress in genetic testing technology, the time taken to diagnose MDs has significantly shortened, helping to significantly reduce the cost of medical treatment for patients.". It is suggested that the medical costs that would be reduced be specified, that is, avoiding the cost of those tests, evaluations and treatment not necessary when confirming the diagnosis, avoiding the diagnostic odyssey that some of these patients suffer.

**Response:** Thank you for your suggestion. We fully agree with your opinion and have included further text on **page 12, lines 222–225** of the revised manuscript.

- Try to explain the following phrase in a simpler way for your better understanding

: "While the overall healthcare insurance coverage rate for this group of patients was 95.2%, the reimbursement rate for MDs before diagnosis was 27.29%, 195 which decreased to 15.16% after diagnosis."

**Response:** We sincerely apologize for the unclear expression of this sentence, which has been revised on **pages 10–11, lines 194–202**: “ *The overall healthcare insurance coverage rate for this patient group reached 95.2%; however, the average reimbursement amount per patient in terms of direct medical costs is extremely low, at only 27.29% before diagnosis and decreasing to 15.16% after diagnosis. This is mainly because the initial healthcare insurance reimbursement rate in China for rare diseases such as MDs is not high. Moreover, most patients concurrently seek medical treatment elsewhere, which further reduces the reimbursement rate. In addition, some tests, such as gene sequencing and metabolic screening of blood and urine, along with certain treatments not covered by healthcare insurance, lead to increased economic burdens on patients’ families.* ”

- When talking about gene therapy in mitochondrial diseases, it is important to mention that these treatments will increase direct expenses, hence the importance of calculating the indirect expenses that could potentially improve thanks to these future treatments.

**Response:** I completely agree with your comment. In response to your suggestion, we have added further details on **pages 13, lines 254–258** of the Discussion section in the revised manuscript.

- It is suggested that a table be added for greater understanding of the questionnaire

**Response:** We sincerely apologize for omitting the questionnaire. In response to your valuable suggestion, we have incorporated this as Supplementary Materials in the revised manuscript for resubmission.

- Table 1: How do you explain that more than 90% of patients have no family history?

**Response:** We apologize for the inaccurate description concerning family history. In this article, the family history only pertained to whether siblings had similar diseases. In line with your suggestion, in the revised manuscript, we have changed ‘family history’ to ‘*Siblings with similar diseases*.’

**Reviewer #2**: The manuscript by Xu and colleagues describes the economic costs of mitochondrial disease in Chinese children. Data from a total of 102 patients were included in the study. Data were collected by a questionnaire completed by the families and validated by neurologists. Families were contacted again directly if there were any uncertainties. In principle, this study is valuable and can form the basis for better advice and care for affected families.

I noticed the following points:

1. Line 227-229 "The highest annual economic cost after diagnosis was the treatment cost, which increased from 20.50% to 48.82% before diagnosis, whereas the examination cost decreased from 34.83% to 3.87% before diagnosis."

I have two questions here:

1.1 Shouldn't it say "after diagnosis" rather than "before diagnosis" in both cases?

1.2 What do the percentages refer to? To the total costs?

**Responses:**

1.1 We apologize for the unclear expression. The sentence has been corrected in the revised manuscript.

1.2 The percentages here refer to the proportion of the total economic cost. In response to the two questions you have raised, we have modified this sentence in the manuscript, as follows:

“ *Among the annual economic costs after diagnosis, treatment costs accounted for the highest proportion of the total economic cost, rising from 20.50% before diagnosis to 48.82% after diagnosis, while the examination costs decreased from 34.83% before diagnosis to 3.87% after diagnosis.* ” (**page 12, lines 236–239**)

2. Lines 229-233 (treatment). How do patients obtain the treatment listed? Are the substances prescribed by the doctors? The substances mentioned are not evidence-based for all MDs. Later, it is also mentioned that ineffective medication is also taken in China because there is obviously still hope that it will be effective.

Maybe a major message of the present study could be that ineffective treatment should be avoided since this comprises the major cost factor after the genetic diagnosis has been established.

**Response:** Thank you for your comments and questions. In this study, the information on the listed treatment medications was provided to patients by clinical doctors, based on guidelines for MDs [PMID: 25503498; PMID: 24700433; PMID: 33105273]. Some of these medications were obtained through prescriptions, while many more were purchased through over-the-counter channels in the form of health products. As you have pointed out, although these drugs are commonly used in the clinical treatment of MDs, evidence-based data vary depending on different MD subtypes.

In China, cultural factors such as patients' keen expectations for treatment effects and traditional medical concepts have led to the phenomenon that patients still insist on receiving relevant treatments even when doctors have informed them that certain medications may be ineffective. Moreover, a considerable number of these drugs fall into the category of health products and are not covered by medical insurance, which undoubtedly significantly increases the treatment costs. We have fully considered this issue and have actively tried to call for the avoidance of treatment methods lacking evidence-based support following a clear genetic diagnosis, so as to reduce the unnecessary economic burden on patients.

3. In the patient cohort 49 patients with MELAS are included. Are these really patients with symptoms such as "stroke-like episodes"? This clinical manifestation is rather rare in pediatric patients. Or are they patients with the most common mitochondrial DNA variants for MELAS (especially m.3243A>G)? If it is mainly patients with m.3243A>G, this should be formulated more clearly.

**Response:** Thank you for your insightful question. All 49 patients with MELAS in this cohort experienced stroke-like episodes.

We apologize for not presenting the genetic information concerning the patients with MELAS. Among the 49 patients with MELAS, 46 had the m.3243A>G mutation, with one patient each having m.3271T>C, m.3252A>G, and m.3256C>T mutations, respectively. This information has been included on **pages 8, lines 143–144** of the revised manuscript.

4. Line 235-238: I think the word "is" should be added after "… clinical trials of MD drugs [is] gradually increasing, …"

**Response:** We apologize for this grammatical error, and the amendment has been made in the revised manuscript.
